# CCR5 signaling promotes lipopolysaccharide-induced macrophage recruitment and alveolar developmental arrest

**DOI:** 10.1038/s41419-021-03464-7

**Published:** 2021-02-15

**Authors:** Ze Chen, Xiaohua Xie, Na Jiang, Jianhui Li, Lei Shen, Yongjun Zhang

**Affiliations:** 1 Department of Neonatology, Xinhua Hospital, Shanghai Jiao Tong University School of Medicine, 1665 Kong Jiang Road, 200092 Shanghai, China; 2 Department of Neonatology, Shanghai Children’s Hospital, Shanghai Jiao Tong University, 355 Lu Ding Road, 200062 Shanghai, China; 3 Shanghai Institute of Immunology, Shanghai Jiao Tong University School of Medicine, Building No. 5(West Area), No. 280 South Chongqing Road, 200025 Shanghai, China

**Keywords:** Interleukins, Bacterial infection

## Abstract

The pathogenesis of bronchopulmonary dysplasia (BPD), involves inflammatory, mechanisms that are not fully characterized. Here we report that overexpression of C-C chemokine receptor 5 (CCR5) and its ligands is associated with BPD development. Lipopolysaccharide-induced BPD rats have increased CCR5 and interleukin-1β (IL-1β) levels, and decreased alveolarization, while CCR5 or IL-1β receptor antagonist treatments decreased inflammation and increased alveolarization. CCR5 enhances macrophage migration, macrophage infiltration in the lungs, IL-1β levels, lysyl oxidase activity, and alveolar development arrest. CCR5 expression on monocytes, and its ligands in blood samples from BPD infants, are elevated. Furthermore, batyl alcohol supplementation reduced CCR5 expression and IL-1β production in lipopolysaccharide-exposed rat lungs. Moreover, receptor-interacting kinase 3 (RIP3) upstream regulator of CCR5-cultured RIP3^−/−^ macrophages exhibited partly blocked lipopolysaccharide-induced CCR5 expression. We conclude that increased CCR5 expression is a key mechanism in BPD development and represents a novel therapeutic target for treatment.

## Introduction

Bronchopulmonary dysplasia (BPD), the most common chronic lung disease among infants, is characterized by premature alveolar developmental arrest^1,2^. The development of BPD is associated with an inflammatory response with elevated growth factor and cytokines levels^3^. Macrophages, which play an important role in the inflammatory process, have been implicated in BPD, as macrophage counts remain elevated in infants that develop the disease^4,5^. Furthermore, tracheal aspirates from preterm infants with BPD not only have higher macrophage counts, but also have increased concentrations of inflammatory cytokines such as interleukin (IL)-10, IL-6, IL-8, macrophage inflammatory protein-1α (MIP-1α/CCL3), and IL-1β^6^. Experimental evidence points to IL-1β being the primary mediator of the changes observed during BPD^7,8^. Nevertheless, although several reports suggest that the excessive production of cytokines and chemokines by pulmonary macrophages contributes to the persistent lung inflammation and decreased alveolarization in BPD, the mechanisms and nature of macrophage recruitment remain to be elucidated^4,5^.

Animal models to study BPD have often used intra-amniotic lipopolysaccharide (LPS) administration to inhibit postnatal lung development. This results in fewer and larger alveoli in lungs, which mimics the lung morphology of BPD in premature human infants^9–12^.

Seeking to identify the factors contributing to abnormal lung morphogenesis in BPD, we used RNA sequencing (RNAseq) analysis to characterize the gene expression profiles in LPS-treated primary macrophages. The analysis indicates that the expression of C-C chemokine receptor 5 (CCR5) and its ligand, C–C chemokine ligand 3 (CCL3), was significantly upregulated in LPS-treated cells. CCR5 is a G-protein coupled receptor known to regulate the immune response by interacting with any of its three chemokine ligands (CCL3, CCL4, and CCL5)^13^. Previous studies have elucidated that CCR5 promotes targeted migration of immune cells, leading to undesirable inflammatory side effects^14,15^. High expression levels of CCR5 in Vδ2 T cells contributes to the pathogenesis of rheumatoid arthritis^16^. In patients with COVID-19, inflammatory macrophages showed a significantly higher expression of the CCL2, CCL3, CCL20, and IL-1β^17^. Ishida et al.^18^ reported that deficiency of either the CCL3 or CCR5 gene markedly attenuated bleomycin-induced lung fibrosis and reduced intrapulmonary macrophage infiltration. In contrast, CCR5 knockout mice showed increased mortality rates related to the pulmonary inflammatory response to influenza A virus infection^19^. These conflicting data suggest that CCR5 is critical in mediating innate immune responses against infections or inflammation; however, presently, there is no data demonstrating whether CCR5 signaling could contribute to the etiology of BPD or how LPS regulates CCR5.

Here we demonstrated that the expression of CCR5 and its ligands was significantly increased in infants with BPD and also in rat models of BPD. CCR5 inhibition partially restored the alveolar development impaired by LPS in vitro. CCR5 and its ligands enhanced macrophage migration to lungs, which overproduced IL-1β and led to lysyl oxidase (LOX) overactivation. We also demonstrated that CCR5 overexpression was partly stimulated by receptor-interacting kinase 3 (RIP3) through NF-κB signaling. These results indicate that CCR5 and its ligands may be involved in the key pathophysiological mechanisms in BPD and as such may be novel therapeutic targets for BPD.

## Materials and methods

### Animals

Rip3flox/flox mice were obtained from Dr. Sudan He (Soochow University, Suzhou, Jiangsu, China). Macrophage-specific Rip3 knockout (Rip3LKO) mice were generated by crossing LyzCre and Rip3flox/flox mice. Timed-pregnancy Sprague-Dawley rats were provided by the Shanghai Laboratory Animal Center, Shanghai, China.

### Neonatal rat model of BPD

The Animal Care and Use Committee at Xinhua Hospital, Shanghai, China approved all mouse and rat protocols. We used a previously established animal model of BPD^10,11^. Briefly, at 16.5 days of gestation (term: 22 days), pregnant rats were prepared to receive intra-amniotic injection and were randomly assigned to the saline control, LPS, LPS with batyl alcohol (BTA), LPS with D-ala-peptide T-amide (DAPTA), LPS with anti-IL-1β antibody (anti-IL-1β), LPS with GSK872 (RIP3 kinase inhibitor) or IL-1β group. The saline control group received 5 μL of normal saline per amniotic sac, the LPS group while the LPS, LPS with BTA, LPS with DAPTA, LPS with anti IL-1β, LPS with GSK872 and IL-1β groups received 1 μg of LPS (Escherichia coli 055: B5; Sigma-Aldrich, St. Louis, MO, USA), 1 μg of LPS and 0.4 μg of BTA (Bachem, Bubendorf, Switzerland), 1 μg of LPS and 1 μg of DAPTA (Tocris Bioscience, Bristol, UK), 1 μg of LPS and 0.05 μg of anti-IL-1β (PeproTech, Rocky Hill, NJ, USA), 2 μg of GSK872 (Selleckchem,Houston, TX, USA), and 0.5 μg of IL-1β (PeproTech), respectively, diluted in 5 μL normal saline per amniotic sac. The day the pups were born was designated as postnatal day 0 (P0). Both male and female rats were used, and animals were killed on P1, P3, and P7 to examine the time course changes for all measurements.

### Rat lung harvest

Anesthesia for all experiments was induced using an intraperitoneal injection of a cocktail containing 100 mg/kg ketamine and 16 mg/kg xylazine.The left lung was inflated using 4% paraformaldehyde for paraffin sectioning. For each time point (P1, P3, and P7), sections were processed for hematoxylin and eosin (H&E) and gomori staining to examine differences in lung architecture using light microscopy.The right lung was immediately frozen in liquid nitrogen and stored at −80 °C.

### Lung morphometry

Three pups (one each at P1, P3, and P7) were selected from each group, and six random non-overlapping fields in one distal lung section per pup were utilized for morphometric examinations. The analysis of lung morphometry was performed as previously described^10^.

### Isolation of alveolar macrophages and bone marrow-derived macrophages (BMDMs)

Alveolar macrophages were isolated from the BALF of P7 rats, and BMDMs were obtained from Rip3LKO mice. BALF was collected using sterile, ice-cold normal PBS with 1% penicillin and streptomycin (10 000U/mL) in aliquots of 1 mL (total volume, 8 mL) and immediately centrifuged to pellet the alveolar macrophages. The pelleted cells were resuspended and cultured at RPMI 1640 (Sigma-Aldrich) with 10% heat inactivated fetal bovine serum (FBS; Gibco, Grand Island, NY, USA) and 1% penicillin and streptomycin.

BMDMs were obtained from Rip3LKO mice. Briefly, bone marrow was flushed from the tibia and femur of each mouse using macrophage medium (RPMI with 10% FBS, 20% L929 conditioned medium, 1% penicillin/streptomycin/glutamine) under sterile conditions, and a single-cell suspension was prepared by gentle pipetting and straining. Then the cells were cultured in macrophage medium with 30 ng/mL M-CSF (R&D Systems, Minneapolis, MN, USA). Cells were allowed to further differentiate for 3 days. BMDMs were either maintained quiescent or treated with either LPS (1 μg/mL) to stimulate Toll-like receptor 4 (TLR4) or T (TNF-α 20 ng/mL; R&D Systems) S (smac minmetic 100 nM; R&D Systems) Z(zVAD 20 μM; Bachem), or pyrrolidine dithiocarbamate (PDTC; 50 μM, Sigma-Aldrich) for 16 h.

### ELISA

The concentration of IL-lβ in supernatant samples from cultured BMDMs, and the production of IL-lβ in the lung tissues, were assessed using an IL-1β ELISA kit (Cat. # 88-6010-22; R&D Systems) according to the manufacturer’s instructions. The concentrations of CCR5 ligands in infant peripheral plasma were assessed using the corresponding ELISA kits (eBioscience, San Diego, CA, USA) according to the manufacturer’s instructions.

### Western blot analysis

All the protein samples were subjected to electrophoresis by SDS-PAGE method and then transferred to PVDF membrane for further immunoblot. The primary antibodies used in this study include: RIP3 antibody (Cat. # ab56164; Abcam, Cambridge, UK; 1:1000), phospho-RIP3 antibody (Cat. # ab56164; Abcam; 1:1000), CCR5 (Cat. # ab65850; Abcam; 1:1000), Tublin (Cat. # ab65850; Abcam; 1:1000), phospho-p65 antibody (Cat. # 3033; Cell Signaling Technology, Danvers, Massachusetts, USA; 1:1000), GAPDH (Cat. # 01404; StemCell Technologies, Vancouver, CA; 1:1000). The blots were developed using a chemiluminescence system (Amersham Pharmacia Biotech, Piscataway, NJ, USA).

### Immunostaining and macrophage phenotyping

After paraffin removal and rehydration, endogenous fluorescence was blocked with 0.1% sodium borohydride. Nonspecific binding was blocked by incubation with 100% goat serum overnight at 4 °C, and then sections were incubated with primary antibodies overnight at 4 °C. The primary antibodies used in this study include: F4/80 (Cat. # sc-377009; Santa Cruz Biotechnology, CA, USA; 1:50), IL-lβ (Cat. # AF-501-NA; R&D Systems; 1:50). After washing, the slides were incubated with a fluorophore-conjugated secondary antibody (Goat Anti-Mouse IgG (H + L, Alexa Fluor 488; Cat. # 115-545-003; Jackson Immuno Research Philadelphia, PA, USA; 1:500, Donkey Anti-Goat IgG (H + L,APC; Cat. # 705-136-147; Jackson Immuno Research; 1:200) for 60 min. Slides were washed and mounted with Fluoromount-G (Southern Biotechnology Associates, Birmingham, AL). All sections were observed under low-power magnification to ensure that all areas of the lung section had been included in the determinations.

### Transwell migration assay

Migration capacity was determined by transwell assay using transwell inserts (Corning Life Science, Kennebunk, ME, USA). For transwell migration assays, 200 µL heated culture medium without FBS was filled in the top chamber, and a noncoated membrane (24-well insert; pore size, 8 μm; Corning, NY, USA) was polymerized in the transwell inserts for 0.5–1 h at 37 °C. Cells (6 × 104, RAW264.7 (RAW264.7 cell line was purchased from American Type Culture Collection (ATCC; http://www.atcc.org/)) or alveolar macrophages were plated in the top chamber and the lower chamber was filled with 600 μL 10% FBS with 6 μL CCL3 (0.1 µg/µl; PeproTech) as a chemoattractant. Cells were incubated for 12 h or 72 h, and those that did not migrate through the pores were removed using a cotton swab. Cells that migrated to the lower surface of the membrane were fixed and stained with 0.1% crystal violet for 20 min. The cells on the bottom of the membrane were counted from five different microscopic fields, and the average number was calculated.

### LOX activity assay

The activation of LOX in lung tissues was assessed using a fluorometric LOX activity assay kit (Cat. # ab112139; Abcam) according to the manufacturer’s instructions.

### Flow cytometry

Peripheral blood mononuclear cells (PBMCs) were collected according to the manufacturer’s instructions (Cat. # 86415; Stem Cell, Vancouver, Canada) and characterized by surface staining with human antibodies against CD14 (Cat. # 555399; BD Bioscience, Franklin Lakes, NJ, USA), CD16 (Cat. # 557744; BD Bioscience) and CD195 (CCR5) (Cat. # 564512; BD Bioscience). Cells were stained for 1 h at room temperature, and then washed in FACS buffer three times. All flow cytometry acquisitions were performed using an LSR II (BD Bioscience).

### Patients and controls

This study was approved by the ethical committee of Xinhua Hospital, Shanghai Jiao Tong University School of Medicine (XHEC-C-2016-016); parents or legal guardians provided signed, written informed consent for participation. Peripheral blood samples were collected from infants with birth weights between 800 and 2000 × *g* born at the neonatal intensive care of Xinhua Hospital, Shanghai Jiao Tong University School of Medicine between 2018 and 2019. BPD was diagnosed according to the criteria proposed by the National Institute of Child Health and Human Development^20^. Infants assigned to one of two groups—infants with BPD (*n* = 7) or premature infants without BPD (*n* = 9)—were recruited for testing CCR5 expression from blood PBMCs. Infants with BPD (*n* = 24) and premature infants without BPD (*n* = 19) were also recruited for measurements of blood levels of CCL3, CCL4, and CCL5.

### Statistical analysis

Data are presented as means ± standard deviations (SDs). The sample sizes used were based on the expected levels of changes and consistency. Statistical significance was reported as appropriate. Student’s t-test was used for statistical comparisons between the means of two groups; for multiple comparisons, one-way analysis of variance was used. SPSS 18.0 (SPSS Inc., Chicago, IL, USA) was used for all analyses. *p* < 0.05 was considered statistically significant.

## Results

### Murine BPD induced by intra-amniotic LPS injection is prevented by a neutralizing anti-IL-1β antibody

**Fig. 1:**
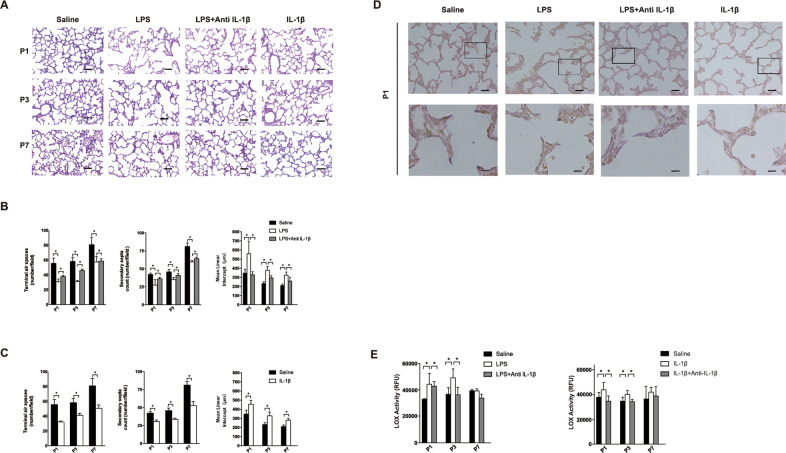
IL-1β mediated arrested effects on alveolarization in the alveolar development phase. **A** Representative lung sections stained with H&E. Scale bar: 50 μm. **B** Quantification of the terminal airspace, secondary septa, and MLI of lung tissues from P1, P3, and P7 rats treated with LPS and anti-IL-1β antibody. Data are expressed as mean ± SD (*n* = 6), *p < 0.05. **C** Quantification of the terminal airspace, secondary septa, and mean linear intercept of lung tissues from P1, P3, and P7 rats treated with IL-1β. Data are expressed as mean ± SD (*n* = 6), *p < 0.05. **D** Representative lung sections stained with gomori (Amplified). Top: Scale bar: 50 μm. Bottom: Scale bar: 25 μm. Treatment with either LPS or IL-1β resulted in abnormal elastin deposition in the lung septal, whereas adding anti-IL-1β antibody normalized elastin. **E** Quantification of LOX enzymatic activity in whole lung lysate of P1, P3, P7 rats treated with LPS and anti-IL-1β antibody. Data are expressed as mean ± SD (*n* = 7), *p < 0.05. Qua*n*tification of LOX enzymatic activity in whole lung lysate of P1, P3, and P7 rats treated with IL-1β and anti-IL-1β antibody. Data are expressed as mean ± SD (*n* = 7), *p < 0.05.

Pregnant rats were injected with LPS or saline in the amniotic sac at E16.5. Lung tissue samples from the LPS-injected group displayed alveolar simplification, as indicated by enlarged alveoli with decreased terminal airspace, decreased secondary septa, and increased mean linear intercept (MLI). We observed similar effects when rat amniotic sacs were injected with IL-1β, but cotreatment with anti-IL-1β antibody and LPS increased terminal airspace and secondary septa counts and decreased MLI (Fig. 1a–c).

As we reported previously, LPS led to abnormal localization of elastin in the lungs of newborn rats^21^. In this study, we also assessed the activity of LOX, which plays a key role in regulating the stability of the extracellular matrix (ECM)^22^. As expected, LPS or IL-1β treatment resulted in disorganized elastin fiber networks in the developing septa—mostly in primary septa with a few in secondary septa—whereas treatment with anti-IL-1β antibody effectively alleviated the abnormal localization of elastin at P1 (Fig. 1d). Thereafter, LOX activity was examined in the injured developing lung. LOX activity was increased in the lungs of LPS-exposed or IL-1β-exposed pups compared with that in the control group. In contrast, an anti-IL-1β antibody prevented stimulus-induced LOX activation (Fig. 1e).

### Macrophages increase in number and are the primary source of IL-1β in LPS-exposed rat lungs

**Fig. 2:**
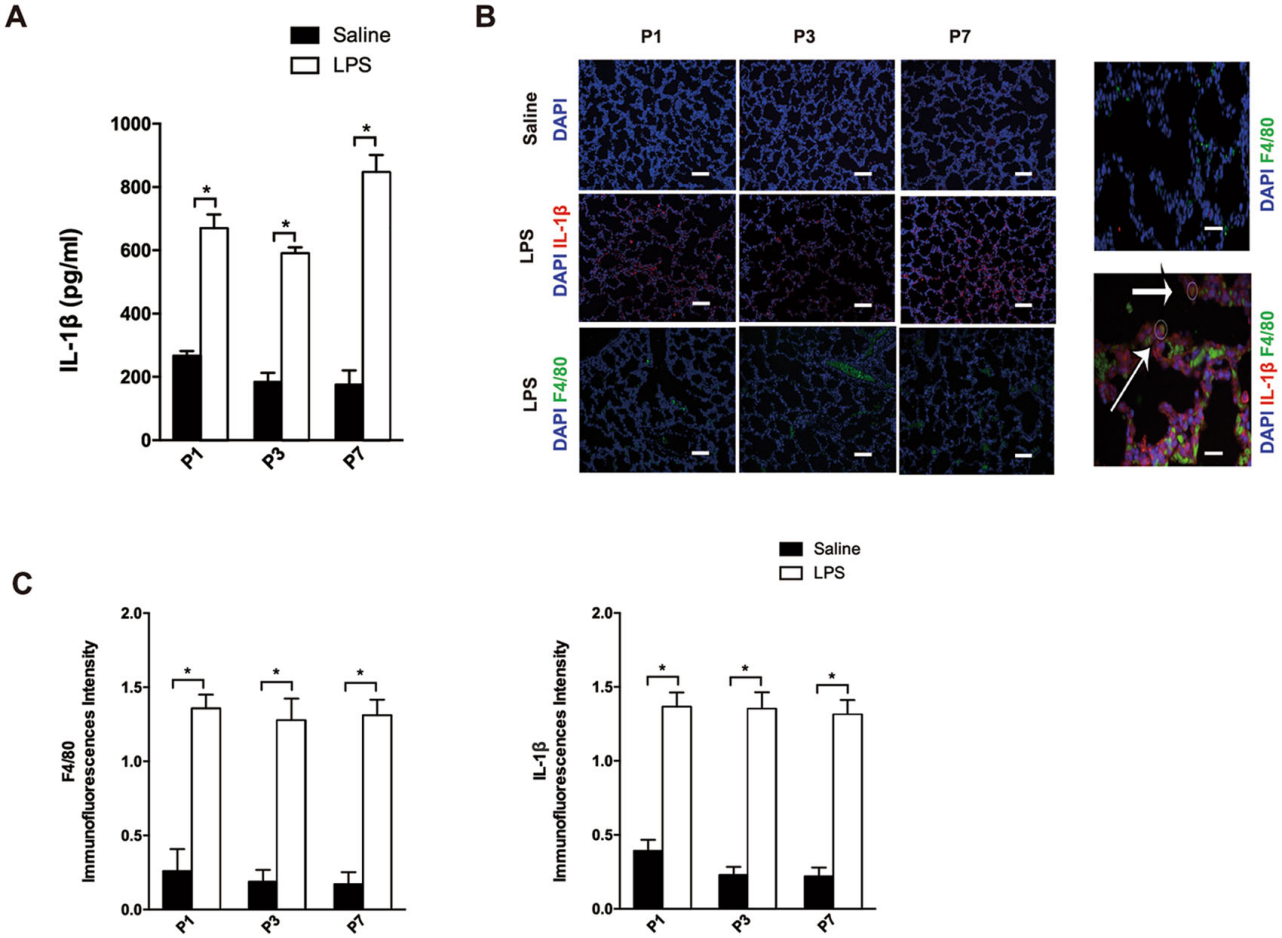
Macrophages increased in number and were the primary sources of IL-1β in antenatal LPS–treated rats. **A** IL-1β protein levels in rat lung tissues. Data are expressed as mean ± SD (*n* = 7), *p < 0.05. **B** Representative images of lung tissues immunostained for F4/80 and IL-1β. Scale bar: 50 μm. Representative images of lung tissues with IL-1β secreted from alveolar macrophages of antenatal rats (Arrows). Scale bar: 25 μm. **C** Quantitation of F4/80 and IL-1β fluorescence intensity. Data are expressed as mean ± SD (*n* = 7), *p < 0.05.

IL-1β levels in rat lungs were increased in the LPS injection group by 2.5-fold, 3.2-fold, and 4.8-fold at P1, P3, and P7, respectively (Fig. 2a). To examine whether the upregulation of IL-1β is mainly confined to macrophages, we examined its expression in lung tissue using immunofluorescence analysis. LPS treatment caused alveolar macrophage infiltration of the lungs in pups at P1, P3, and P7 pups (Fig. 2b). Few macrophages were detected in the lungs in the control group. In addition, LPS administration increased the levels of macrophage IL-1β compared with control treatment. LPS-induced IL-1β elevation was mainly observed in macrophages as identified by co-staining for macrophages and IL-1β. Moreover, the level of IL-1β paralleled the number of macrophages in the lungs (Fig. 2c). These data suggest that macrophages represent the major source of alveolar IL-1β in our model of LPS-induced BPD.

### CCR5 facilitates alveolar macrophage migration and IL-1β overproduction to promote alveolar development arrest

As macrophage and cytokine IL-1β played a key role in the BPD development^7,8^, we used RNAseq analysis to characterize the gene expression profiles in LPS-treated primary macrophages. GO analysis showed abundant gene expression changes in LPS-treated primary macrophages compared to normal macrophages that correlate with IL-1β. We found the top ten significantly elevated genes (Table S1). Among them, GBP5 and NOD2 gene expression was significantly increased. However, according to previous studies, NOD2 is more highly related to intestinal inflammation than macrophages^23^, while GBP5 is mainly induced by interferon gamma (IFN-γ) and is involved in innate immunity against a wide variety of microbial pathogens^24^. However, CCR5s are expressed predominantly on monocytes and macrophages^25^, and may drive the recruitment of these cells to the lungs where they release IL-1β. Therefore, we hypothesized that blocking CCR5 may effectively prevent disease manifestation.

**Fig. 3:**
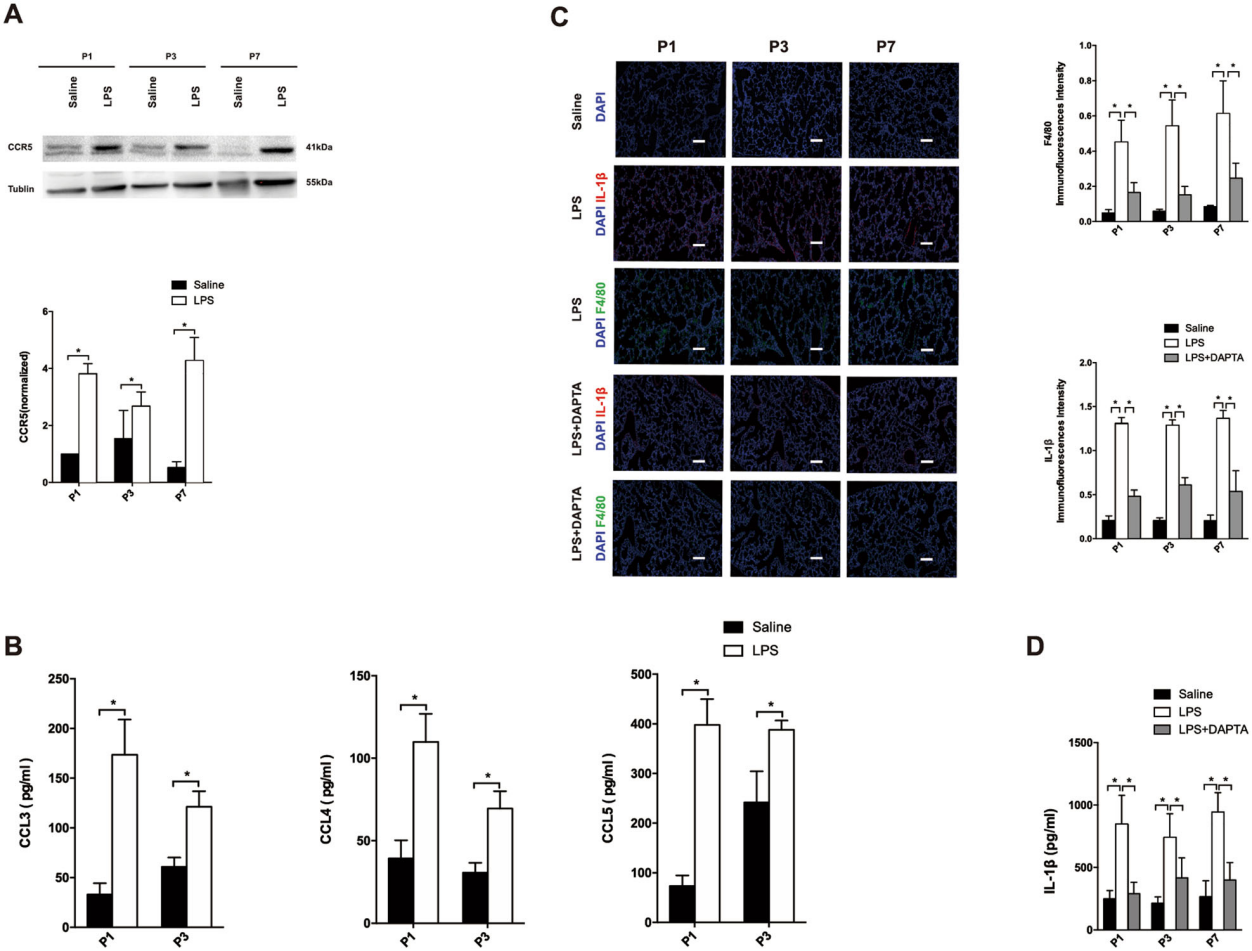
CCR5 inhibition reduced the numbers of macrophages in the lungs and suppressed IL-1β overproduction in the rat model of BPD. **A** Representative immunoblot probed for CCR5 in whole lung lysates of P1, P3, and P7 rats. Data are expressed as mean ± SD (*n* = 6), *p < 0.05. **B** CCL3, CCL4, and CCL5 levels in rat lung tissues. Data are expressed as mean ± SD (*n* = 8), *p < 0.05. **C** Representative images of lung tissues immunostained for F4/80 and IL-1β. Scale bar: 50 μm. Quantitation of F4/80 and IL-1β fluorescence intensity. Data are expressed as mean ± SD (*n* = 6), *p < 0.05. **D** IL-1β protein level in rat lung tissues. Data are expressed as mean ± SD (*n* = 8), *p < 0.05.

To test this hypothesis, we firstly analyzed the expression of CCR5 in the antenatal LPS-treated rat lungs. As shown in Fig. 3a, expression of CCR5 increased significantly at P1, P3, and P7. Moreover, levels of CCL3, CCL4, and CCL5 were also elevated in the LPS-injected group at P1 and P3 (Fig. 3b). We next found that treatment with DAPTA, a CCR5 antagonist^26^, suppressed LPS-induced production of excess IL-1β in the lungs (Fig. 3d). DAPTA treatment also reduced the number of macrophages in the LPS group to levels comparable to those in the control group (Fig. 3c).

**Fig. 4:**
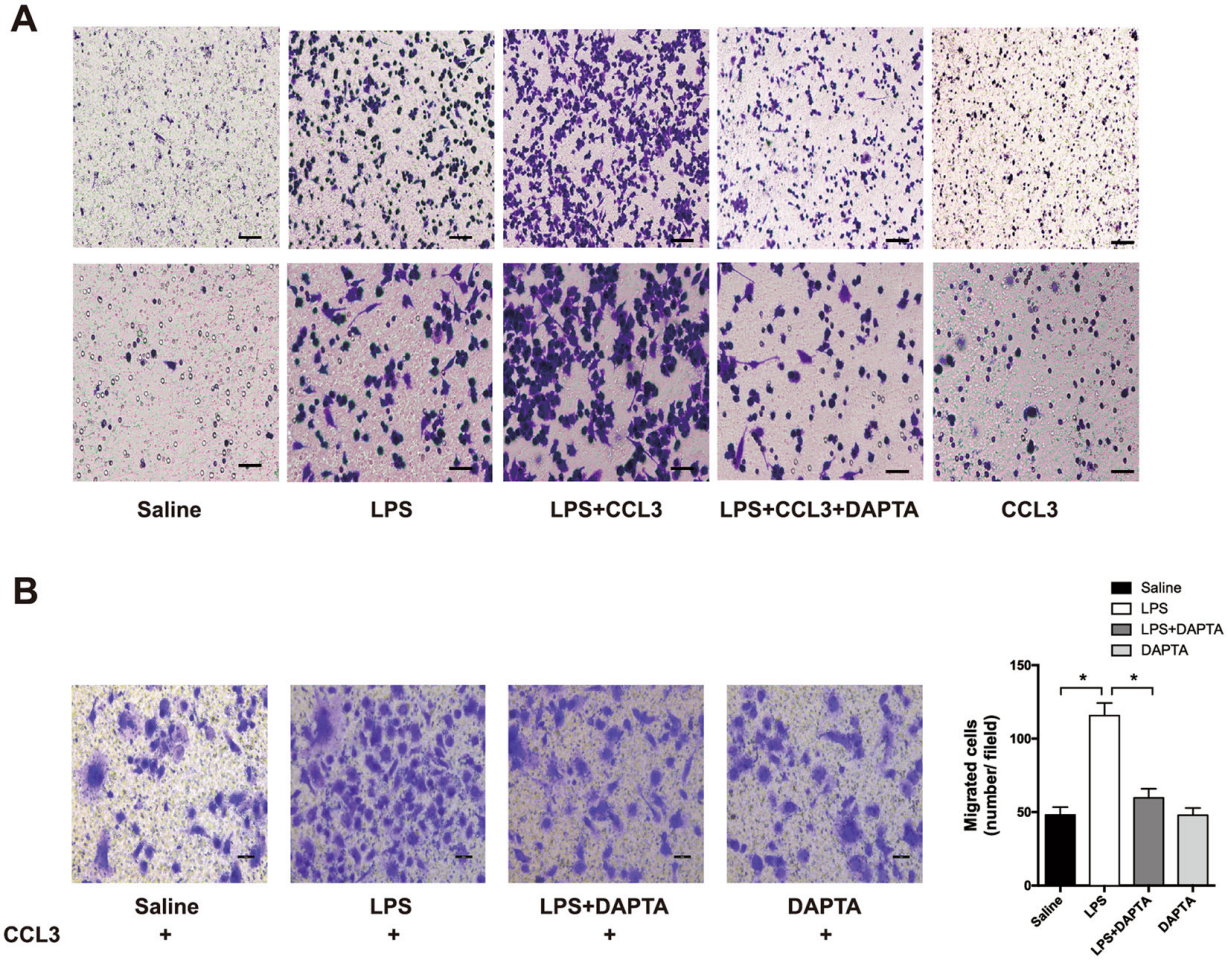
Enhanced migration of macrophages induced by LPS toward CCL3. **A** Representative images of migrating RAW264.7 cells treated with LPS (1 µg/µl) and/or CCL3 (0.1 µg/µl), and DAPTA (1 µg/µl) for 12 h. Top: Scale bar: 100 μm. Bottom: Scale bar: 50 μm. **B** Representative images of migrating macrophages from BALF after treatment with LPS (1 µg) and/or DAPTA (1 μg) for 72 h. Scale bar: 50 μm. Quantification of transwell migrated macrophages. Data are expressed as mean ± SD (*n* = 7), *p < 0.05.

In order to ensure that the enhanced chemotactic response of monocytes was mediated via CCR5, migration assays were performed in vitro and in vivo. Cultured RAW264.7 cells treated with LPS exhibited significant migratory capacity compared to those treated with PBS, as indicated by a marked increase in the number of migrated cells. The RAW264.7 LPS treatment group also migrated in greater numbers toward CCL3 than cells from PBS control group. In contrast, the migratory response of RAW264.7 cells from the LPS group toward CCL3 was completely inhibited by DAPTA (Fig. 4a).

We further collected macrophages from the BALF of BPD rats and analyzed their migration ability. Consistent with the results of RAW264.7 cells in the LPS treatment group, there was increased migration of macrophages from BPD lungs toward CCL3 when compared with controls (Fig. 4b).

**Fig. 5:**
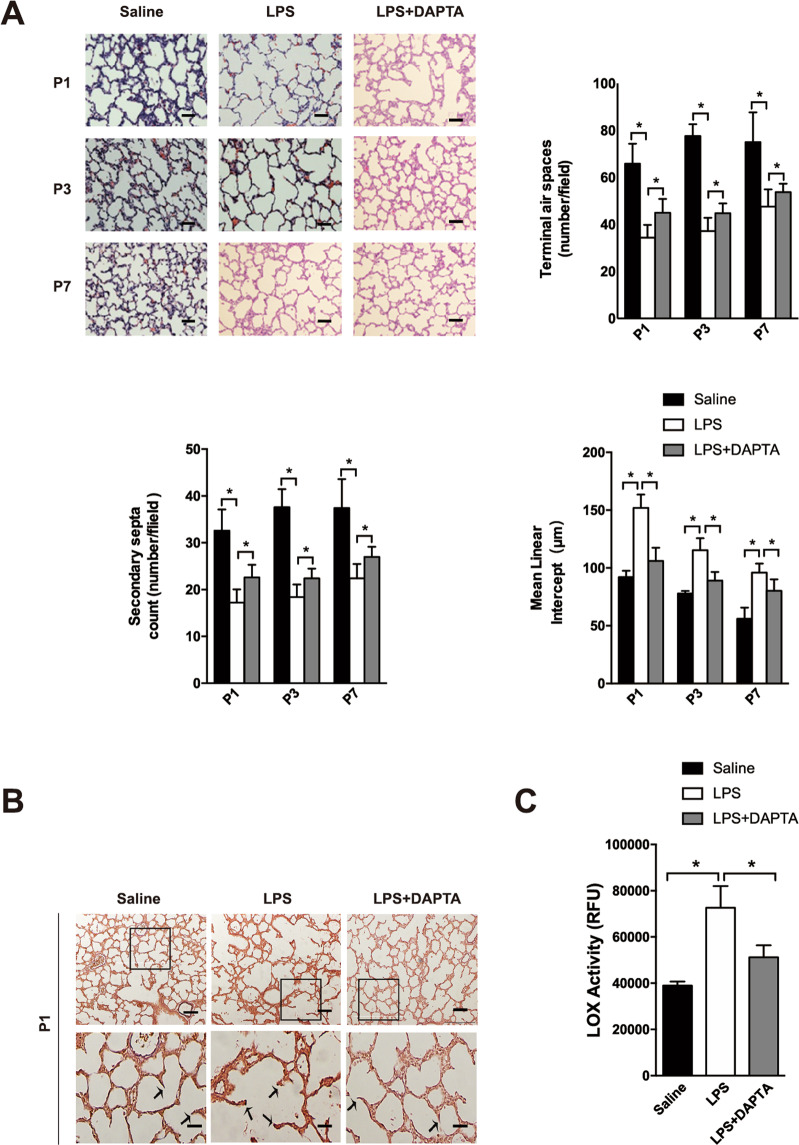
CCR5 mediated arrested effects on alveolarization in antenatal LPS–treated rats. **A** Representative lung sections stained with H&E. Quantification of the terminal airspace, secondary septa, and MLI. Scale bar: 50 μm. Data are expressed as mean ± SD (*n* = 6), *p < 0.05. **B** Representative lung sections stained with gomori (Arrows). Top: Scale bar: 50 μm. Bottom: Scale bar: 25 μm. **C** Quantification of LOX enzymatic activity in whole lung lysate of P1 rats. Data are expressed as mean ± SD (*n* = 7), *p < 0.05.

Next, pregnant rats were injected with LPS and DAPTA in the amniotic sac. Pups receiving LPS and DAPTA cotreatment showed significantly reduced LOX activity, attenuated abnormal localization of elastin, and improved alveolarization compared with those in the LPS alone group, although these parameters did not completely normalize to the levels in the saline group (Fig. 5).

### RIP 3 acts as one of the upstream regulators of CCR5

Although the current model predicts that the proinflammatory function of RIP3 is largely due to its role in necroptosis, it is noteworthy that RIP3 can either enhance NF-κB activity or facilitate pro-IL-1β processing in macrophages^27,28^. NF-κB regulates the expression of inflammatory mediators that recruit monocytes, drives differentiation to macrophages, and directs macrophage cell fate determination^29^. Hence, it is possible that RIP3 facilitates inflammation through CCR5-mediated molecular patterns.

**Fig. 6:**
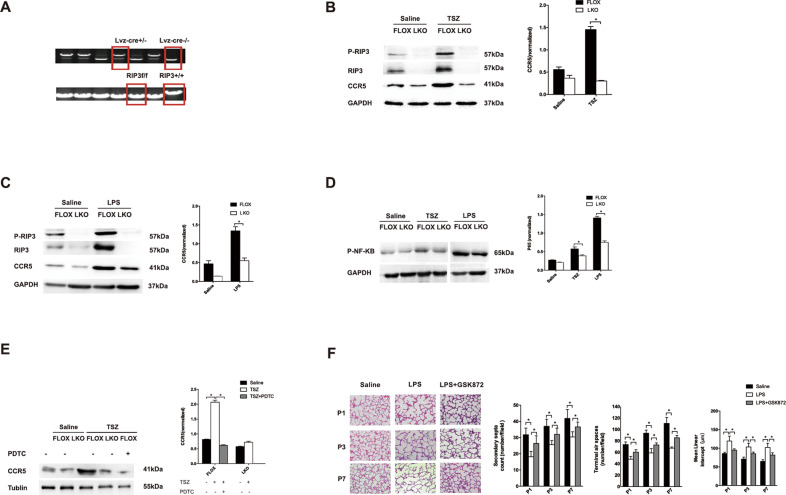
RIP3 regulates CCR5 through NF-kB. **A** Genotyping of Rip3LKO mice. **B** Representative immunoblot probed for CCR5 in whole BMDM lysate from Rip3LKO stimulated with TSZ (20 ng/mL TNF-α, 100 nM smac minmetic, 20 μM zVAD) for 16 h and corresponding quantified data. Data are expressed as mean ± SD (*n* = 6), *p < 0.05. **C** Representative immunoblot probed for CCR5 in BMDM lysates from Rip3LKO mice stimulated with LPS (1 μg/mL) for 16 h and corresponding quantified data. Data are expressed as mean ± SD (*n* = 6), *p < 0.05. **D** Representative immunoblot probed for NF-kB in BMDM lysate from Rip3LKO mice stimulated with TSZ or LPS at the indicated conditions for 16 h and the corresponding quantified data. Data are expressed as mean ± SD (*n* = 6), *p < 0.05. **E** Representative immunoblot probed for CCR5 in BMDM lysate from Rip3LKO mice stimulated with TSZ at the indicated condition and PDTC (50 μM) for 16 h and the corresponding quantified data. Data are expressed as mean ± SD (*n* = 6), *p < 0.05. **F** Representative lung sections stained with H&E. Scale bar: 50 μm. Quantification of the terminal airspace, secondary septa, and MLI of lung tissues from P1, P3, and P7 treated with LPS and GSK872. Data are expressed as mean ± SD (*n* = 6), *p < 0.05.

As shown in Fig. 6a–c, expression of CCR5 was increased in RIP3+/+ BMDMs induced by LPS or the RIP3 activator TSZ. By contrast, TSZ-induced expression of CCR5 was severely inhibited in RIP3−/− BMDMs. However, unlike TSZ, LPS-induced expression of CCR5 was slightly increased in RIP3−/− BMDMs, indicating that LPS-induced CCR5 expression through mechanisms other than by influencing RIP3. In addition, TSZ-induced NF-κB activation was diminished in RIP3−/− BMDMs. Moreover, the NF-κB inhibitor PDTC blocked TSZ-induced CCR5 expression (Fig. 6d, e). Altogether, this indicates that RIP3 is an upstream regulator that stimulates CCR5 expression through NF-κB. Furthermore, pups which received LPS and GSK872 (RIP3 kinase inhibitor) following their injection into the amniotic sac of pregnant rats showed increased terminal airspace and secondary septa counts but decreased mean linear intercept (Fig. 6f).

### BTA inhibits CCR5 and IL-1β production and alleviates the simplification of the lung alveolar in BPD rat model

**Fig. 7:**
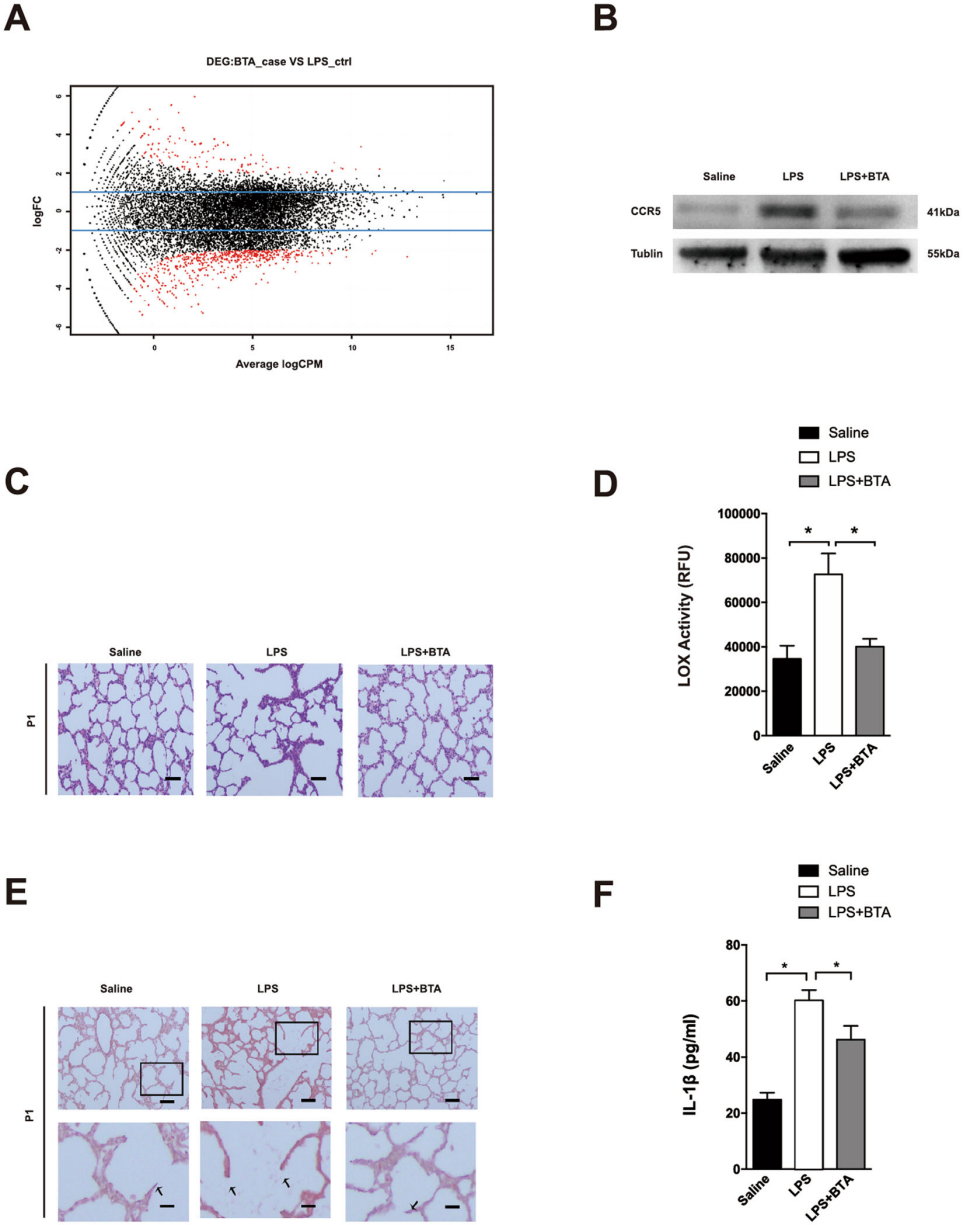
BTA abated CCR5 function and IL-1β production to alleviate the simplification of the lung alveolar in BPD rat model. **A** RNAseq analysis in LPS and BTA treated primary macrophages. **B** Representative immunoblot probed for CCR5 in whole lung lysate of P1 rats. **C** Representative lung sections stained with H&E. Scale bar: 50 μm. **D** Treatment with LPS increased LOX activity; adding BTA normalized LOX activity. Data are expressed as mean ± SD (*n* = 7), *p < 0.05. **E** Representative lung sections stained with gomori (Arrows). Top: Scale bar: 50 μm. Bottom: Scale bar: 25 μm. **F** IL-1β levels in rat lung tissues. Data are expressed as mean ± SD (*n* = 8), *p < 0.05.

Natural products (NPs) are important sources of clinical drugs due to their structural diversity and biological prevalidation, particularly in the field of cancerous and infectious diseases^30–32^. We screened the anti-inflammatory activities of several NPs including BTA, *Eclipta alba* (EAP), Vitamin D, and *Astragalus* polysaccharide (APS) in LPS-treated primary macrophages using RNAseq analysis. We quantified CCR5 gene expression by calculating the reads per kilobase million (RPKM). Our RNAseq results show that EAP upregulated CCR5 gene expression compared to LPS-treated primary macrophages (RPKM: 32.440 vs. 8.467). BTA, Vitamin D, and APS could downregulate CCR5 expression compared to LPS-treated primary macrophages. (RPKM: 0.627, 2.585, and 4.321, respectively, vs. 8.467). Of these, BTA caused the greatest reduction in CCR5 with a 13.5-fold decrease as compared to Vitamin D (3.3-fold decrease) and APS (2.0-fold decrease). Thus, we considered that BTA had the most significant effect. BTA is a known component of deep-sea shark liver oil and has anti-inflammatory activities^33^. Similar results were also observed in antenatal LPS-treated rat lungs (Fig. 7a, b). Next, LPS with or without BTA was injected into the amniotic sac of pregnant rats at E16.5. Compared with LPS treatment alone, cotreatment with BTA and LPS increased terminal airspace and secondary septa counts but decreased MLI at P1 (Fig. 7c). Moreover, BTA also reduced LOX activity, attenuated abnormal localization of elastin, and decreased IL-1β levels (Fig. 7d–f).

### CCR5 and its ligands are upregulated in patients with BPD

**Fig. 8:**
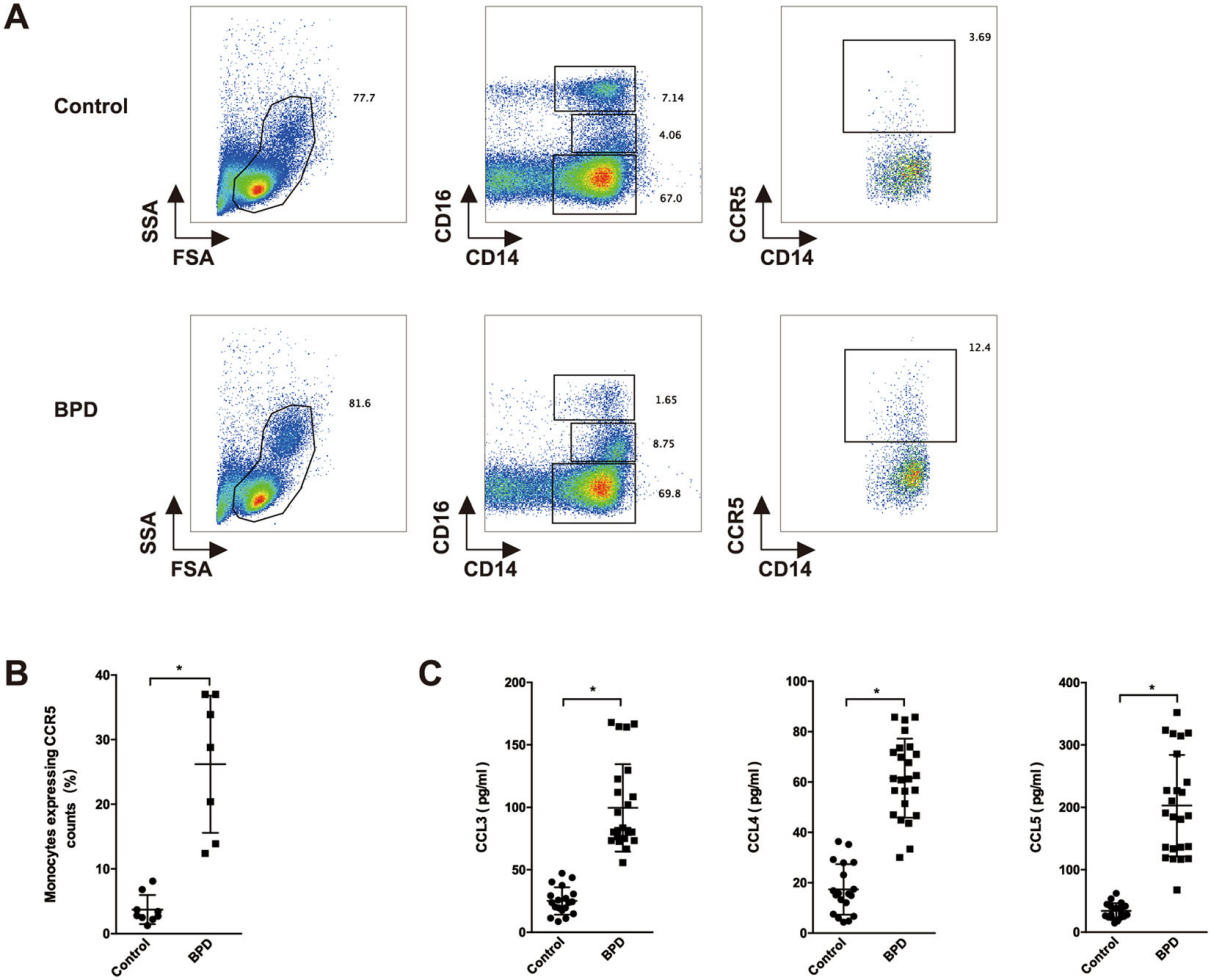
CCR5 and its ligands were upregulated in infants with BPD. **A** Representative flow plot of CCR5-expressing CD14^+^CD16^+^ monocytes in control infants (*n* = 9) and infants with BPD (*n* = 7). **B** Quantitative analysis of the percentage of CCR5-stained CD14^+^CD16^+^ monocytes. **C** Plasma CCL3, CCL4, and CCL5 levels in control infants (*n* = 19) and infants with BPD (*n* = 24). *p < 0.05.

To further assess the level of CCR5 expression in BPD, cells were analyzed by flow cytometry for CCR5 expression on CD14 + CD16 + monocytes from nine healthy donors and seven infants with BPD. No differences were found in gestational age (220.7 ± 11.40 vs. 192.1 ± 6.13 days, *p* = 0.062), sex (female: 4 vs. 2, male: 5 vs. 5, *p* = 0.515), and multiple pregnancy (multiple pregnancy: 1 vs. 2, singleton pregnancy: 8 vs. 5, *p* = 0.374) between the study and control groups. We observed significant upregulation of CCR5 expression in infants with BPD (Fig. 8a, b). To confirm this finding, we collected peripheral blood samples from 19 healthy infants and 24 infants with BPD to assess the abundance of the CCR5 ligands CCL3, CCL4, and CCL5 (Fig. 8c). No differences were found in terms of gestational age (213.2 ± 3.66 vs. 199.3 ± 3.71 days, *p* = 0.013), birth weights (1366.0 ± 62.24 vs. 1192.0 ± 77.61 g, *p* = 0.099), sex (female: 10 vs. 9, male: 9 vs. 15, *p* = 0.321), and multiple pregnancy (multiple pregnancy: 9 vs. 7, singleton pregnancy: 10 vs. 17, *p* = 0.220) between the study and control groups. Consistent with the flow cytometry analysis, serum levels of CCL3, CCL4, and CCL5 were significant higher in samples from infants with BPD than in those from healthy infants (Fig. 8c).

## Discussion

In this study, we found a critical role of CCR5 signaling in the pathogenesis of BPD and demonstrated that the expression of CCR5 and its ligands was significantly increased in infants with BPD, as well as in an LPS-induced rat model of BPD, which had never been previously reported. Importantly, increased CCR5 levels promote the aggregation of macrophages in the lungs and induce the production of IL-1β. Blocking CCR5 decreased inflammation and improved alveolarization, thereby ameliorating the BPD phenotype. Furthermore, we determined that RIP3 could regulate the expression of CCR5. Lastly, we found that batyl alcohol supplementation could reduce CCR5 expression and IL-1 β production in LPS-exposed rat lungs.

Several studies have reported the association of inflammation with the subsequent development of BPD in preterm infants^3,34^. IL-1β, a key initiator of inflammation, has a complex activation system and has been implicated in several inflammatory disorders^35^. Elevated levels of IL-1β in amniotic fluid and postnatally are also associated with the development of BPD^36^, and an IL-1 receptor antagonist alleviated the abnormal lung structure in animal models of BPD^37^. In agreement with these data, we found that IL-1β production was increased at P1, P3, and P7 in the LPS-treated group. Furthermore, double immunostaining data demonstrated increased accumulation of macrophages in the lungs of BPD rats exposed to LPS and that these inflammatory cells are the major source of IL-1β. Hence, elevated IL-1β levels might contribute to two possible mechanisms in our model. The first is the increased expression of IL-1β in macrophages. Stimulation of TLR4 with LPS can lead to activation of the transcription factor NF-κB and then induce the expression of proinflammatory molecules such as IL-1β^38^. The second is that IL-1β overproduction is a consequence of increased macrophages numbers.

We also found that LOX activity increased in the lungs of the LPS-induced group, and this upregulation was driven, at least in part, by excessive IL-1β. These observations are important because LOX proteins play a key role in regulating the stability of ECM. Any perturbation to the formation and remodeling of matrix structures would be expected to affect the development of the immature lung^22^. For example, thickened, tortuous, and disorganized elastic fibers have been observed in patients with BPD, and excessive production and accumulation of elastin has also been reported in animal models of BPD^39,40^. Thus, elevated levels of IL-1β might underlie the aberrant activity of LOX proteins in BPD, leading to abnormal localization of elastin.

It is known that macrophage numbers increase in BPD, and also that CCR5 is expressed predominantly on monocytes and macrophages. An important finding of this study is that the expression of CCR5 and its ligands was increased in infants with BPD and in a rat model of BPD. Such expression patterns suggest that these chemokine receptors could alter both innate and adaptive immune responses important in BPD pathogenesis. In addition, we observed a heightened chemotactic response of macrophages from BPD rats toward CCL3, suggesting that enhanced macrophage numbers in BPD-affected lungs are a consequence of increased recruitment of monocytes from the circulation, since there is little proliferation within the lung^41^, although there is a possibility that the increased lung macrophages in BPD were transformed from resident alveolar macrophages (rAMs). It was reported that rAMs may transdifferentiate to a novel population of cells with characteristics of macrophages in BPD models exposed to hyperoxia^4^. Moreover, increased BPD monocyte migration may be related to the increase in the levels of CCR5 ligands such as CCL3 in the blood. In obese white adipose tissue inflammation, the CCL5-CCR5 axis triggered adhesion and transmigration of blood monocytes and exerted antiapoptotic properties on WAT macrophages^42^. In addition, CCL3/CCR5 blockage reduced monocyte chemotaxis to chronic obstructive pulmonary disease sputum supernatant^43^. Clinical trials that have used CCR5 antagonists include studies to prevent graft versus host disease and cancer metastasis^44,45^. The most frequently used CCR5 antagonist is maraviroc, a U.S. Food and Drug Administration–approved drug to treat patients infected with CCR5-tropic HIV-1 in combination with other antiretroviral agents^46^. Therefore, it may be possible to test CCR5 antagonists in clinical studies in patients with BPD in the near future.

RIP3-dependent necrosis is widely believed to contribute to several detrimental pathologies. Conversely, RIP3 is profoundly important for inflammation, in part through regulating NF-κB activity^47^. Here, we found that TSZ-induced expression of CCR5 was severely inhibited in RIP3−/− BMDMs, and the NF-κB inhibitor PDTC blocked TSZ-induced CCR5 expression. Thus, RIP3 could enhance NF-κB activity, resulting in CCR5 overexpression. However, CCR5 levels were slightly elevated in RIP3−/− BMDMs after LPS treatment, suggesting that RIP3 activation is not necessary for LPS-induced CCR5 expression. LPS and its receptor TLRs lead to the production of proinflammatory cytokines, as well as activation of the downstream signaling pathway of the transcription factor NF-κB^48^. So, it is possible that LPS stimulates CCR5 expression through NF-κB but without RIP3. In addition to the signaling pathway mentioned above, other transcription factors like GATA, STAT3, and CREB can also activate the CCR5 pathway^49^. In contrast, activated NF-κB can make proIL-1β biologically active. At the same time, IL-1β can also upregulate the activation of NF-κB^50,51^. Consequently, such an inflammatory response likely drives a vicious pathogenic cycle, leading to even more inflammation.

BTA is an important alkylglycerol which has been reported to have anti-inflammatory effects^52–54^. Oh et al^55^. found that peripheral blood granulocytes were significantly elevated, and plasma IgG and IgM levels were significantly higher, in pups with alkylglycerol supplementation than in controls. In our study, we found that BTA supplementation reduced CCR5 expression and IL-1β production in BPD lungs. This may be a new mechanism by which BTA can restrain inflammation, and thus BTA may be a new target drug for BPD.

In summary, our results establish a new molecular mechanism pertaining to disrupted lung morphogenesis following intrauterine inflammation. We demonstrate that CCR5 signaling promotes LPS-induced macrophage recruitment and contributes to arrested alveolar development as observed in BPD. These results suggest that this pathway may be a promising target for BPD treatment.

## Supplementary information

Table S1. Ten significantly elevated gene expressions related with IL-1β.
