# Supplementary material for: CCR5 signaling promotes lipopolysaccharide-induced macrophage recruitment and alveolar developmental arrest
Source: Cell Death Dis. 2021 Feb 15;12(2):184. doi: 10.1038/s41419-021-03464-7 (PMC7883330; doi:10.1038/s41419-021-03464-7)
Supplement: Supplementary file 1 — Table S1. Ten significantly elevated gene expressions related with IL-1β. [file 41419_2021_3464_MOESM1_ESM.docx]

**Table S1.** **Ten significantly elevated gene expressions related with IL-1β.**

| **Gene Name** | **PBS_control_RPKM** | **LPS_case_RPKM** | **Times** |
| --- | --- | --- | --- |
| Gbp5 | 0.1849 | 14.3613 | 77.67063277 |
| **Ccl3** | **32.8734** | **1074.0345** | **32.67184106** |
| Nod2 | 0.4488 | 11.0919 | 24.71457219 |
| **Ccr5** | **0.3735** | **8.467** | **22.66934404** |
| Mefv | 0.2394 | 3.4357 | 14.3512949 |
| Casp4 | 0.1596 | 2.2051 | 13.81641604 |
| Ifnar1 | 26.2035 | 206.1998 | 7.869170149 |
| Lgals9 | 18.2595 | 8.1638 | 0.447098771 |
| Gstp1 | 1234.5315 | 158.6405 | 0.128502594 |

RPKM, reads per kilobase million
